# Increased production of humoral factor and PD-L1 expression induced by cytokine stimulation play a key role in the immunosuppressive effects of stem cells derived from human exfoliated deciduous teeth

**DOI:** 10.1016/j.bbrep.2025.102295

**Published:** 2025-10-02

**Authors:** Shin Tsunekawa, Makoto Kato, Kenta Iwasaki, Yuko Miwa, Yusuke Hayashi, Takako Izumoto, Akihito Yamamoto, Tatsuhito Himeno, Masaki Kondo, Takaaki Kobayashi, Hideki Kamiya, Kohei Ishiyama

**Affiliations:** a Division of Diabetes, Department of Internal Medicine, Diabetes Center, Aichi Medical University, Nagakute, Japan; b Department of Diabetes, Endocrinology and Metabolism, Gifu University Graduate School of Medicine, Gifu, Japan; c Department of Kidney Disease and Transplant Immunology, Aichi Medical University, Nagakute, Japan; d Department of Oral and Maxillofacial Surgery, Tokoname City Hospital, Tokoname, Japan; e Department of Histology and Oral Histology, Institute of Biomedical Sciences, Tokushima University Graduate School, Tokushima, Japan; f Department of Renal Transplant Surgery, Aichi Medical University, Nagakute, Japan

## Abstract

**Background:**

Recently, the efficacy of mesenchymal stem cells (MSC) targeting transplant immunosuppression has been reported, and their application to islet transplantation is also expected. However, since the stability of functional manifestation of MSC remains unclear, we have investigated the stability and efficacy of cytokine-stimulated stem cells from human exfoliated deciduous teeth (SHED) in the human immune system in order to establish a safety clinical application.

**Methods:**

SHED were stimulated with TNF-α, IL-1β, and IFN-γ, which were elevated post-transplant in the liver. Flow cytometry was used to analyze surface antigen expression. Human peripheral blood mononuclear cells (PBMC) were co-cultured with stimulated SHED directly or indirectly to assess PBMC proliferation. Cytotoxicity assay evaluated PBMC-induced damage to induced pluripotent stem (iPS) cell-derived human pancreatic beta-like cells. ELISA measured immunomodulatory factor secretion.

**Results:**

Programmed death-ligand 1 (PD-L1) expression was assessed, and PBMC were co-cultured with stimulated SHED in the presence of anti-PD-L1 antibody. SHED were also reaggregated with dissociated human pancreatic islets to generate islet-like organoids, and insulin secretion was measured. Stimulated SHED showed minimal change in cell surface markers but significantly inhibited PBMC proliferation and cytotoxicity, Stimulated SHED produced elevated levels of immunosuppressive factors and expressed PD-L1. The immunosuppressive effect was partially inhibited by inhibiting cell contact between SHED and PBMC or blocking the PD-1/PD-L1 pathway. Furthermore, insulin secretion was enhanced in reaggregated human pancreatic islets with SHED.

**Conclusions:**

We demonstrated that the use of SHED in its activated state effectively suppresses immune response and maintains graft function at the time of islet transplantation.

## Highlights

• Addressing immune responses in islet transplantation remains the critical challenge in improving transplantation outcomes.
• Immunosuppressive effects of SHED and BM-MSC were evaluated in the human immune system.
• SHED-induced immunosuppression, mediated by humoral factors and cellular contact, was stronger than that by BM-MSC.
• SHED also improved insulin secretion in reaggregated human islets, suggesting a promising adjunct for islet transplantation.

## 1 Introduction

Islet transplantation alone is preferred over pancreas transplantation in patients with type 1 diabetes who have not yet developed renal failure [1]. This shift has been attributed to significant clinical experience, developments in islet isolation techniques, and immunosuppressive therapies accumulated over the past two decades since the implementation of the Edmonton Protocol [2]. Islet transplantation offers the advantage of being minimally invasive; however, its outcomes, such as insulin independence rates, are still lower than those of pancreas transplantation [3]. The primary factors for suboptimal transplantation outcomes include islet graft loss due to hypoxia [4], mechanical damage [5], and immune-mediated reactions, particularly those involving innate immune responses [[6], [7], [8]]. Particularly, addressing immune responses remains a critical challenge in improving transplantation outcomes.

Instant blood-mediated inflammatory reaction (IBMIR) is characterized by the initial activation of the coagulation and complement systems after transplanting islets into the portal vein, followed by rapid binding and activation of platelets [9]. IBMIR is thought to be the initial trigger of immune response immediately after transplantation and is marked by the release of inflammatory cytokines, such as IFN-γ, TNF-α, and IL-1β [8,10,11]. These cytokines activate mesenchymal stem cells (MSC) and induce the production of various soluble factors, including prostaglandin E2 (PGE2), TGF-β, and indoleamine 2,3-dioxygenase (IDO) [12]. We previously reported that pre-activated MSC, which was incubated with IFN-γ, TNF-α, and IL-1β prior to co-transplantation with islets, significantly suppressed the immune response after intraportal islet transplantation and markedly enhanced islet graft survival compared to naive MSC in a mouse model [13].

MSC have been studied with respect to immunosuppression; however, they are unstable in their properties and difficult to apply in clinical practice. Therefore, we focused on dental pulp stem cells derived from human exfoliated deciduous teeth (SHED), which originate from the cranial neural crest and exhibit marker characteristics of both embryonic and MSC [14]. We have reported that soluble factors secreted by SHED directly protect and promote β-cell propagation [15], as well as angiogenesis [16] and neurite outgrowth in neurons [17], and the effects of SHED treatment are more pronounced than those of MSC derived from bone marrow (BM-MSC). The impact of SHED on islet transplantation, including its effect on post-transplant immune responses, remains unknown. However, BM-MSC have demonstrated the potential to suppress allograft rejection in several experimental models of islet transplantation [8,13,18].

To develop an innovative approach for islet transplantation, we investigated the efficacy of SHED in immunomodulation using human peripheral blood mononuclear cells (PBMC) and pancreatic islets. If the simultaneous administration of SHED improves the treatment efficiency of islet transplantation, islet transplantation will become more widespread, owing to the reduction of islet grafts per recipient.

## 2 Materials and methods

### 2.1 Cell culture

Human BM-MSC were obtained from Lonza, whose datasheet indicated that CD105, CD44, CD90, and CD73 were >90 % positive and that CD14, CD34, and CD45 were expressed at <10 %. MSC were cultured in DMEM (Gibco) supplemented with 10 % FBS and antibiotics (100 U/mL penicillin and 100 mg/mL streptomycin; Wako). MSC passaged 6–8 times were used for the experiments.

Exfoliated deciduous teeth were collected for clinical purposes from donors aged 6–12 years at Nagoya University and Tokushima University Hospital. Written informed consent was obtained from all donors as well as their parents or legal guardians after confirming their willingness to participate. Participation in this study was entirely voluntary and no financial or material compensation was provided. This study was approved by the Institutional Ethical Committees of Nagoya University (Permit number H-73) and Tokushima University Hospital (Permit number 3268) and performed in accordance with the principles of the Declaration of Helsinki. SHED were collected and cultured as described previously [14]. SHED passaged 10–14 times were used for the experiments.

### 2.2 Stimulation by inflammatory cytokine

MSC and SHED at 80 % confluence were rinsed with PBS and then cultured in serum-free DMEM with 20 ng/mL or 100 ng/mL IFN-γ (Sigma-Aldrich), TNF-α (Sigma-Aldrich), and IL-1β (Sigma-Aldrich), or cultured without cytokines for 24 h. Subsequently, the media were collected, and each dish was rinsed with PBS. After adding prewarmed TrypLE^TM^ Express (Gibco) and incubating the cells at 37 °C for 10 min, the detached cells were collected and used for further experiments.

### 2.3 Flow cytometry analysis

Flow cytometry was performed using The BD LSRFortessa™ X-20 (Becton Dickinson and Company, Franklin Lakes, NJ), and the obtained data was analyzed using FlowJo software (Tree Star Software). The following antibodies were used for analyzing cell surface antigen expression on MSC and SHED: anti-human CD274 (PD-L1)-PE, anti-human HLA-A, B, C-FITC, anti-human HLA-DR-FITC, anti-human CD73-FITC, anti-human CD90-FITC, anti-human CD105-PE/Cyanine7, anti-human CD14-PE, anti-human CD34-biotin, and anti-human CD45-PE (Biolegend).

### 2.4 Measurement of immunosuppressive factors

The concentrations of immunosuppressive factors in conditioned media of MSC and SHED were measured with a human enzyme-linked immunosorbent assay kit (indoleamine 2,3-dioxygenase [IDO; Abcam], TGF-β (Proteintech) and prostaglandin E2 [PGE2; Enzo Life Sciences]) according to the manufacturer's instructions. Especially, PGE2 is a low-molecular-weight compound, so the competitive ELISA method is stable. The ELISA standard consists of a seven-point two-fold dilution series with a range of 39.1 pg/ml to 2500 pg/ml. Since this is a competitive assay, the higher the concentration of PGE2 in the supernatant, the lower the OD value measured at 405 nm. For dilution ratios, the supernatant of MSC was diluted 10-fold for both low and high, while the supernatant of SHED was diluted 100-fold for low and 500-fold for high.

### 2.5 Proliferation assay

Human PBMC from healthy volunteers were isolated from 20 mL of whole blood by density gradient centrifugation using Histopaque-1077 (Sigma-Aldrich). The proliferation of the purified PBMC was evaluated using carboxyfluorescein diacetate succinimidyl ester (CFSE) labeling. CFSE-labeled PBMC were seeded in 96-well flat-bottomed plates (2.0 x 10^5^ cells/well) in AIM-V containing Hepes and anti-CD3/CD28 coated microbeads and were either cultured alone or cocultured with MSC or SHED at a ratio of 10:1 to 640:1. After incubation for 3 days at 37 °C in 5 % CO_2_, the PBMC were identified with anti-human CD3-APC/Cy7, anti-human CD4-APC, and anti-human CD8-PE. Cell proliferation was analyzed using FlowJo software. The mitotic index was calculated as previously reported [19]. The suppression ratio was calculated by defining the mitotic index of the activated T cells as 100.

### 2.6 Cytotoxicity assay

For *in vitro* cellular cytotoxicity assay, human induced pluripotent stem (iPS) cell-derived pancreatic beta-like cells (Cellartis® hiPS Beta Cells; Takara Bio) were labeled according to the manufacturer's instructions using the N-SPC non-radioactive cellular cytotoxicity assay kit (Techno Suzuta). The cells were pulsed with the BM-HT reagent at 37 °C. After washing three times, the cells were seeded in 96-well flat-bottomed plates (5.0 × 10^3^ cells/well). Prior to the assay, PBMC (2.0 × 10^5^ cells/well) were activated with anti-CD3/CD28 microbeads for 2 days. Activated PBMC were loaded into the wells at an effector-to-target ratio of 40:1 and cocultured for 40 min with MSC or SHED (5.0 × 10^3^ cells/well). Next, 25 μL of the coculture supernatant was mixed with 250 μL of Eu solution, and time-resolved fluorescence was measured with a SpectraMax M (MOLECULAR DEVICES, Tokyo, Japan). The intensity of the cytotoxicity of PBMC to iPS cell-derived pancreatic beta-like cells was assessed by calculating the specific cytotoxicity rate, which was calculated as 100 × [experimental release (counts)–spontaneous release (counts)]/[maximum release (counts)–spontaneous release (counts)].

### 2.7 Reaggregation of human pancreatic islets and SHED

Human pancreatic islets were obtained from Prodo Laboratories, and the islets were dispersed with TryPLE Express (ThermoFisher). SHED at 80 % confluence were rinsed with PBS and dissociated with TryPLE Express (ThermoFisher). To generate islet-like organoids, dispersed islet cells (ICs) and SHED were seeded in a 96-well U-bottom plate (1000 cells/well) for 24 h at 37 °C in 5 % CO2. PIM(S) medium (Prodo Laboratories) was used for both the generation and maintenance of islet-like organoids. This medium is specifically formulated for human islet culture and was provided by Prodo Laboratories, which also supplied the human islets used in this study. Islet-like organoids were formed by mixing ICs and SHED at a ratio of 1:1. To form monocellular IC spheroids, dispersed ICs (1000 cells/spheroid) were seeded. The number of ICs was chosen in order to obtain IC spheroids of approximately 150 μm diameter, i.e., the size of an “islet equivalent” (IEQ). Cell aggregation and spheroid formation were observed under a microscope.

### 2.8 Insulin measurement from islet-like organoids with SHED

Islet-like organoids were incubated for 30 min with Krebs-Ringer buffer containing 2.8 mmol/L glucose. Thereafter, five size-matched islet-like organoids were collected from each plate and then incubated in 100 μL of buffer containing 2.8 or 16.7 mmol/L glucose for 30 min. The islet-like organoids were homogenized in an ice-cold acid-ethanol solution (0.18 M HCl in 75 % ethanol) and centrifuged. The concentrations of insulin released into the medium and cellular insulin content were measured by ELISA using a commercial kit (Mercodia), and insulin secretion was normalized to the cell number.

### 2.9 Statistical analysis

Data are expressed as the mean ± standard deviation. Statistical analyses were performed using a one-way analysis of variance with the Bonferroni test for post-hoc comparisons. Statistical significance was set at p < 0.05. All statistical analyses were performed using EZR software (Saitama Medical Center, Jichi Medical University, Saitama, Japan) [20], a graphical user interface for R (The R Foundation for Statistical Computing).

## 3 Results

### 3.1 Stability of stem cells against inflammatory cytokine

**Fig. 1:**
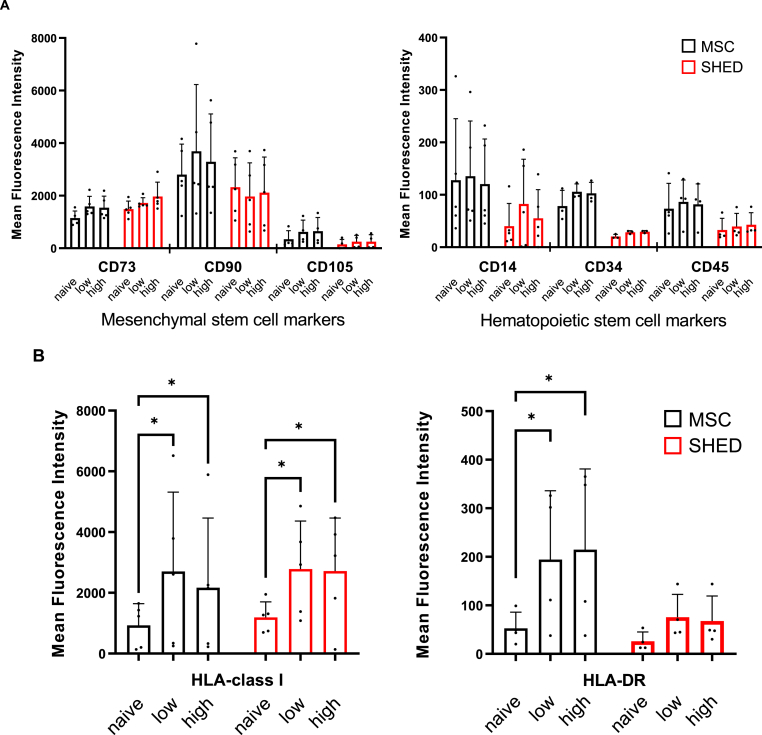
Flow cytometry was performed to assess cell-surface antigens of unstimulated and stimulated MSC and SHED. MSC and SHED were stimulated with inflammatory cytokines TNF-α, IL-1β, and IFN-γ (low; 20 ng/mL or high; 100 ng/mL, respectively). (A) Changes in the expression of mesenchymal stem cell markers and hematopoietic stem cell markers. (B) Changes in the expression of HLA. Data are shown as mean ± standard deviation. Experiments were performed in more than triplicate. ∗p < 0.05 vs non-stimulated control.

MSC and SHED were incubated in serum-free DMEM with or without the combination of 20 ng/mL or 100 ng/mL IFN-γ, 20 ng/mL or 100 ng/mL TNF-α, and 20 ng/mL or 100 ng/mL IL-1β, for low dose or high dose cytokine stimulating condition, respectively. Low dose cytokine stimulation mimics the IBMIR environment, meanwhile high dose conditions were examined for cell stability. Compared with naive cells, cytokine stimulation caused little change in the expression of phenotypic markers of stem cells, such as CD73, CD90, and CD105, in both MSC and SHED. Additionally, the surface markers expressed on hematopoietic stem cells remained low in both tissue stem cells and were less expressed in SHED than in MSC (Fig. 1A). The cytokine stimulation increased the expression of HLA-class I in both MSC and SHED to the same extent. Regarding HLA-DR, no significant changes were observed in naive and stimulated SHED, but not in MSC, indicating that the properties of SHED were more stable against cytokine stimulation than those of MSC (Fig. 1B). Notably, MSC are a heterogeneous population, and the possibility that they contain antigen-presenting cells cannot be ruled out; however, the possibility of this being the case in SHED is minimal.

### 3.2 Immunosuppressive effect of BM-MSC and SHED on PBMC

**Fig. 2:**
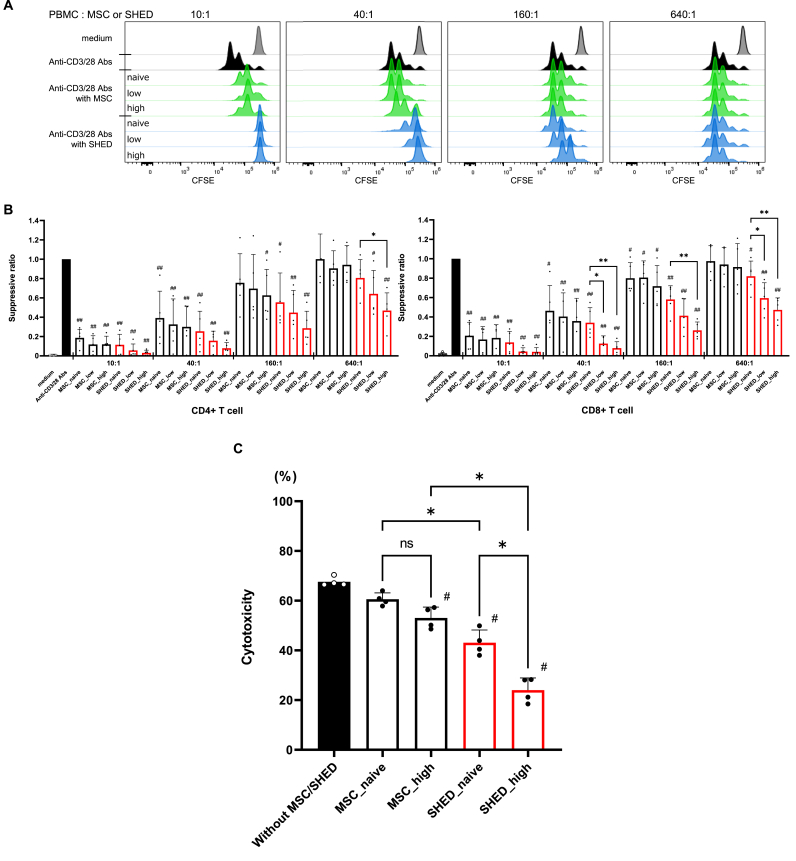
*In-vitro* T cell proliferation assay. CFSE-labeled purified human PBMC were activated by anti-CD3/anti-CD28 coated beads and were either cultured alone or cocultured with MSC or SHED at a ratio of 10:1 to 640:1 for 3 days. (A) Representative determination of PBMC proliferation by flow cytometry. (B) CD4^+^ and CD8^+^ T cell proliferation were analyzed by calculating the suppression ratio. Data are shown as mean ± standard deviation. Experiments were performed 5 times. #p < 0.05, ##p < 0.001 vs without MSC or SHED, ∗p < 0.05, ∗∗p < 0.01 vs. non-stimulated control. (C) Non-radioactive cellular cytotoxicity assay. PBMC and MSC or SHED were cocultured with anti-CD3/anti-CD28 coated beads and then loaded into the wells with human iPS cell-derived pancreatic beta-like cells in them. MSC and SHED were stimulated with 100 ng/mL of TNF-α, INFγ, and IL-1β. The intensity of cytotoxicity of PBMC to human iPS cell-derived pancreatic beta-like cells was assessed by calculating the specific cytotoxicity rate. Data are shown as mean ± standard deviation. N = 4, and experiments were performed in triplicate. #p < 0.001 vs. without MSC or SHED, ∗p < 0.001. MSC_naive, MSC without cytokine stimulation; MSC_low, MSC stimulated with low cytokine dose (20 ng/mL); MSC_high, MSC stimulated with high cytokine dose (100 ng/mL); SHED_naive, SHED without cytokine stimulation; SHED_low, SHED stimulated with low cytokine dose (20 ng/mL); SHED_high, SHED stimulated with high cytokine dose (100 ng/mL).

Immunomodulation by MSC has been reported in several diseases and tissues [21]. Additionally, previous studies have demonstrated that the exposure of MSC to cytokines, mimicking the IBMIR environment, enhances their immunomodulatory effects in a mouse model [22]. To evaluate their immunosuppressive effects on human PBMC, we assessed CFSE-stained cell proliferation using MSC and SHED, either unstimulated or cytokine-stimulated. Compared to monocultured PBMC, including CD4^+^ and CD8^+^ T cells, activated by anti-CD3/CD28 antibody, the suppression ratio, of which the lower score indicates more suppression, in coculture with MSC or SHED was significantly lower in both cytokine concentration and cell number of tissue stem cells-dependent manner, indicating that MSC and SHED suppressed the cell proliferation of PBMC. The suppression ratio of CD4^+^ T cells in coculture with MSC and SHED stimulated by high dose cytokine (MSC_high and SHED_high) were 0.120 ± 0.083 and 0.031 ± 0.038 (10:1 ratio of PBMC to MSC or SHED), 0.300 ± 0.216 and 0.079 ± 0.059 (40:1 ratio of PBMC to MSC or SHED), 0.627 ± 0.268 and 0.286 ± 0.176 (160:1 ratio of PBMC to MSC or SHED), and 0.943 ± 0.198 and 0.469 ± 0.182 (640:1 ratio of PBMC to MSC or SHED), respectively. Similarly, the suppression ratio of CD8^+^ T cells in coculture with MSC_high and SHED_high were 0.185 ± 0.136 and 0.041 ± 0.043 (10:1 ratio of PBMC to MSC or SHED), 0.358 ± 0.234 and 0.079 ± 0.066 (40:1 ratio of PBMC to MSC or SHED), 0.717 ± 0.213 and 0.263 ± 0.086 (160:1 ratio of PBMC to MSC or SHED), and 0.915 ± 0.241 and 0.473 ± 0.124 (640:1 ratio of PBMC to MSC or SHED), respectively (Fig. 2B).

Especially, SHED stimulated with high cytokine concentrations completely suppressed the division of mitotically activated PBMC when the PBMC to SHED ratio was 10:1, effectively preventing activation. Regarding the strength of the suppressive effect, SHED demonstrated significantly greater potency than MSC, as the suppression ratio in coculture with SHED was much lower compared to MSC. Moreover, the suppressive effect of MSC was completely lost when the ratio of MSC to PBMC was 1:640, whereas SHED maintained their suppressive effect at the same ratio. These data suggest that SHED may be more effective than MSC in suppressing PBMC function.

Subsequently, we examined the effects of MSC and SHED on the immune response of PBMC to human iPS cell-derived pancreatic beta-like cells**.** Coculture with MSC or SHED significantly suppressed PBMC cytotoxicity to human iPS cell-derived pancreatic beta-like cells**.** Coculture with SHED significantly suppressed PBMC cytotoxicity compared to coculture with MSC, and this suppressive effect was remarkably enhanced by cytokine stimulation (Fig. 2C). These findings highlight the superior immunosuppressive capacity of SHED over MSC, particularly under cytokine-stimulated conditions, suggesting their potential as an effective immunomodulatory agent for mitigating immune responses in transplantation settings.

### 3.3 Mechanism of immunosuppressive effect BM-MSC and SHED on PBMC

**Fig. 3:**
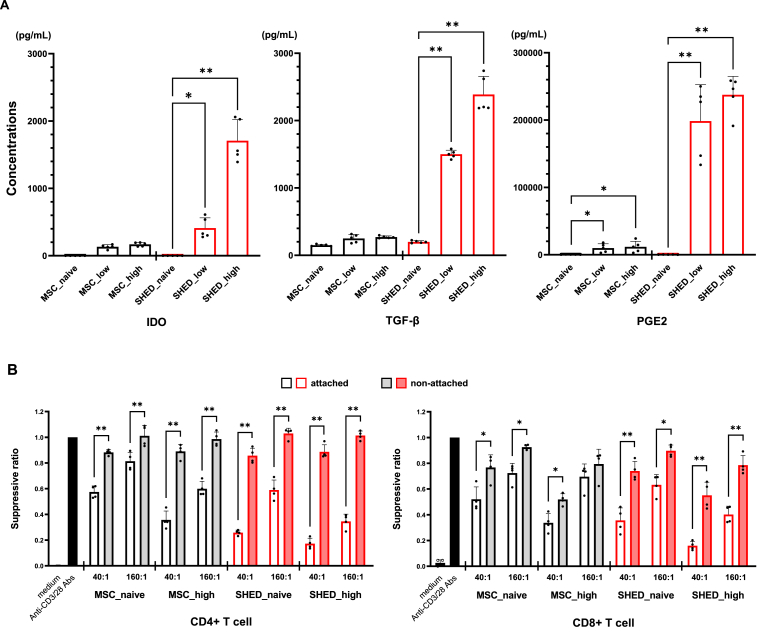
(A) The secretion of the immunosuppressive factors from non-stimulated and stimulated MSC and SHED was evaluated by ELISA. MSC and SHED were stimulated with inflammatory cytokines TNF-α, IL-1β, and IFN-γ (low; 20 ng/mL or high; 100 ng/mL, respectively). Data are shown as mean ± standard deviation. Experiments were performed five times. ∗p < 0.01, ∗∗p < 0.001 vs. non-stimulated control. (B) *In‐vitro* T cell proliferation assay of monolayer cultures. CFSE-labeled purified human PBMC were activated by anti-CD3/anti-CD28 coated beads and were either cultured alone or cocultured with MSC or SHED in 2-dimensional monolayer culture at a ratio of 40:1 and 160:1 for 3 days. CD4^+^ and CD8^+^ T cell proliferation were analyzed by calculating the suppression ratio. Data are shown as mean ± standard deviation. Experiments were performed four times. ∗p < 0.05, ∗∗p < 0.01 vs attached. MSC_naive, MSC without cytokine stimulation; MSC_high, MSC stimulated with high cytokine dose (100 ng/mL); SHED_naive, SHED without cytokine stimulation; SHED_high, SHED stimulated with high cytokine dose (100 ng/mL).

We previously reported that the production of various soluble factors from BM-MSC, such as PGE2 and TGF-β, promoted immunomodulation through NK cell inhibition [13]. Additionally, exposure to cytokines increases the secretion of immunoregulatory factors by BM-MSC [12]. Therefore, we investigated the immunomodulatory factors secreted by BM-MSC and SHED with or without cytokine stimulation to elucidate the mechanism underlying the immunosuppressive effect of both cell types. Cytokine stimulation increased the secretion of representative immunosuppressive factors, including IDO [not detected in either MSC_naive or SHED_naive. 132.5 ± 31.7 pg/mL from MSC stimulated with low cytokine dose (MSC_low), 168.7 ± 28.4 pg/mL from MSC with high cytokine dose (MSC_high), 411.5 ± 151.5 pg/mL from SHED stimulated with low cytokine dose (SHED_low), and 1708.3 ± 312.0 pg/mL from SHED stimulated with high cytokine dose (SHED_high); Fig. 3A], TGF-β (149.5 ± 13.9 pg/mL from MSC_naive, 249.3 ± 60.2 pg/mL from MSC_low, 270.8 ± 16.9 pg/mL from MSC_high, 199.7 ± 17.4 pg/mL from SHED_naive, 1500.7 ± 59.9 pg/mL from SHED_low, and 2387.2 ± 270.5 pg/mL from SHED_high; Fig. 3A), and PGE2 (367.5 ± 128.3 pg/mL from MSC_naive, 9901.1 ± 6393.0 pg/mL from MSC_low, 11803.6 ± 8301.6 pg/mL from MSC_high, 226.0 ± 99.8 pg/mL from SHED_naive, 198540.6 ± 53965.0 pg/mL from SHED_low, and 237567.3 ± 27493.7 pg/mL from SHED_high; Fig. 3A), in a cytokine concentration-depending manner. Moreover, the effect of cytokines on the enhancement of secretion was much greater in SHED than in MSC despite no difference in their secretory capacities under unstimulated conditions. This result suggests that the stronger suppressive effect of SHED on the immune response of PBMC to human iPS cell-derived pancreatic beta-like cells than to MSC may be owing to differences in the secretory capacity of immunoregulatory factors.

Further, we investigated whether the immunosuppressive effects of BM-MSC and SHED on PBMC were dependent on humoral factors. Compared to monolayer cultures where PBMC were attached to MSC or SHED, the suppression ratio was significantly higher in the two-dimensional monolayer culture where PBMC were not attached to MSC or SHED. However, the suppressive effect on PBMC proliferation was sustained at a high ratio of MSC and SHED (Fig. 3B). These data suggest that the suppressive effect of both MSC and SHED on the immunological activity of PBMC was not only because of humoral factors but also cell-to-cell attachment.

**Fig. 4:**
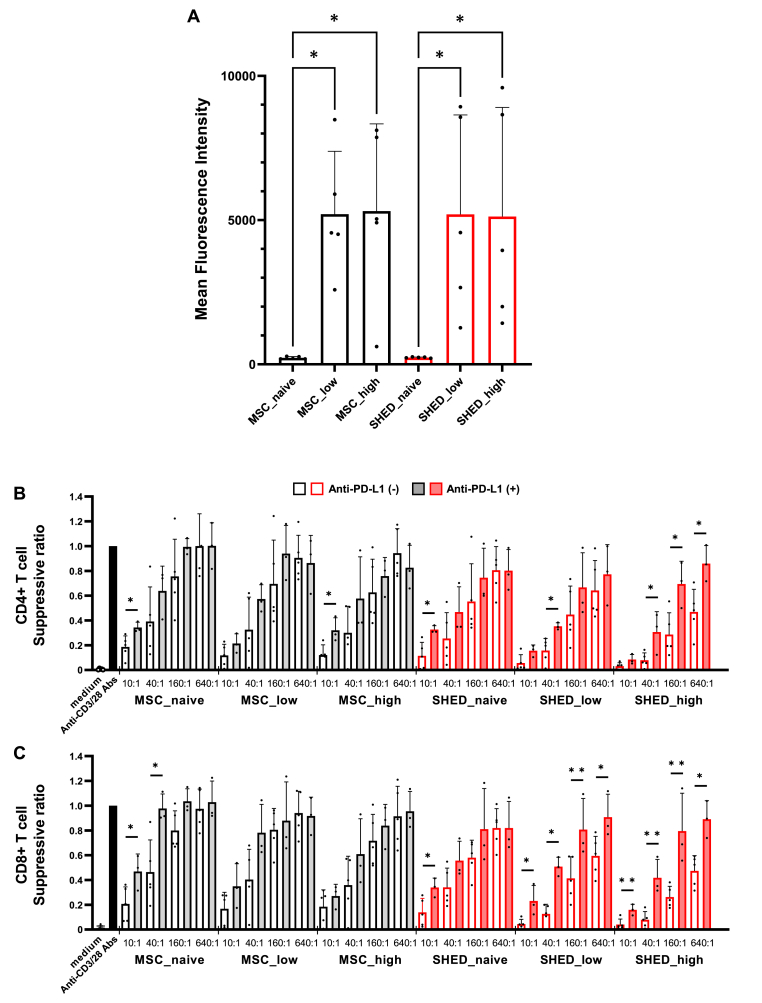
(A) Flow cytometry was performed to assess cell-surface antigens PD-L1 of unstimulated and stimulated MSC and SHED. MSC and SHED were stimulated with inflammatory cytokines TNF-α, IL-1β, and IFN-γ (low; 20 ng/mL or high; 100 ng/mL, respectively). Data are shown as mean ± standard deviation. Experiments were performed five times. ∗p < 0.05 vs. non-stimulated control. (B, C) *In-vitro* T cell proliferation assay. CFSE-labeled purified human PBMC were activated by anti-CD3/anti-CD28 coated beads and were either cultured alone or cocultured with MSC or SHED at a ratio of 10:1 to 640:1 for 3 days with or without anti-PD-L1 blocking antibody. CD4^+^ and CD8^+^ T cell proliferation were analyzed by calculating the suppression ratio. Data are shown as mean ± standard deviation. Experiments were performed in more than triplicate. ∗p < 0.05, ∗∗p < 0.01 vs without anti-PD-L1 blocking antibody control. MSC_naive, MSC without cytokine stimulation; MSC_low, MSC stimulated with low cytokine dose (20 ng/mL); MSC_high, MSC stimulated with high cytokine dose (100 ng/mL); SHED_naive, SHED without cytokine stimulation; SHED_low, SHED stimulated with low cytokine dose (20 ng/mL); SHED_high, SHED stimulated with high cytokine dose (100 ng/mL).

As a mechanism of immunosuppression by cell-to-cell attachment, the interaction between PD-1 and PD-L1 on PBMC is well-known to lead the impairment of function and proliferation of PBMC. Moreover, recent studies have reported that cytokine stimulation enhances PD-L1 expression on the cell surface of various cell types, such as macrophages and mesenchymal stem cells [23,24]. Therefore, we investigated the cell surface expression of PD-L1 in both BM-MSC and SHED. Interestingly, cytokine stimulation led to a prominent enhancement of the cell surface expression of PD-L1 in both MSC and SHED to the same extent (Fig. 4A). Next, we investigated whether the immunosuppressive effects of MSC and SHED on PBMC by cell-to-cell attachment were associated with the cell surface expression of PD-L1. Monolayer cultures with anti-PD-L1 antibody significantly attenuated the suppressive effects of MSC and SHED on PBMC (Fig. 4B and C).

These results suggest that MSC and SHED could suppress the immune activity of PBMC by both humoral factors and cell-to-cell attachment through the interaction of PD-L1, and the immunosuppressive action was enhanced by cytokine stimulation because the cytokines increased the secretion of immunomodulatory factors and cell surface expression of PD-L1.

### 3.4 Insulin secretion from islet-like organoids with islet cells and SHED

**Fig. 5:**
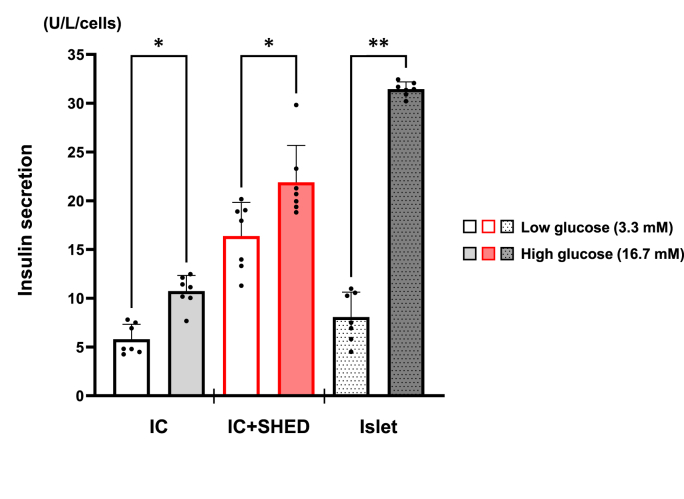
Insulin secretion from islet-like organoids of dispersed human pancreatic islet cells (ICs) with or without SHED and human islets was evaluated via ELISA. One islet-like organoid contains 1000 ICs alone or 500 ICs and 500 SHED. One islet was considered to contain 2000 cells. Insulin secretion was standardized by cell number. Data are shown as mean ± standard deviation. N = 7. ∗p < 0.01, ∗∗p < 0.001. IC, islet-like organoids reaggregated from islet cells only; IC + SHED, islet-like organoids reaggregated from islet cells and SHED; Islet, non-dispersed islets.

These results suggest that SHED might suppress the post-transplant immune response better than BM-MSC do and are more suitable as an adjunctive treatment for islet transplantation. In addition to the immunosuppressive effects of SHED in the present study, we reported cytoprotective effects and enhanced insulin secretion in mouse pancreatic beta cells by humoral factors secreted from SHED [15]. Therefore, we examined the function of islet-like organoids created by reaggregating dispersed pancreatic islet cells and SHED to investigate future clinical applications of SHED in islet transplantation. Islet size is important for transplantation into the liver to prevent blood flow embolization, and islet-like organoids were created by reaggregating dispersed islet cells with SHED in accordance with previous reports on cell counts in islet-like organoids [25]. The reaggregation of dispersed cells showed no significant differences in their morphology or reaggregation time to generate the islet-like organoid between human pancreatic islet cells alone and islet cells with SHED. Compared with non-dispersed islets, insulin secretory capacity was markedly reduced in islet-like organoids reaggregated from solely dispersed human islet cells; however, islet-like organoids incorporating SHED improved the diminished insulin secretory capacity and preserved glucose-dependent insulin secretion (Fig. 5), suggesting that islet-like organoids composed of SHED and islet cells hold potential as a novel technique for islet transplantation in the future.

## 4 Discussion

Overcoming engraftment failure of islet grafts in the early post-transplant period is the most important challenge for successful islet transplantation. It is reported that 20–50 % of transplanted islet grafts fail to survive within a few weeks after transplantation [26]. From an immunological aspect, one of the main causes for this is thought to be IBMIR, which causes grafted pancreatic islet damage due to the activation of blood coagulation factors and the complement system, leading to thrombus formation and activation of the innate immune system immediately after transplantation [8,9]. Therefore, transplanted islet engraftment needs to regulate the specific immune environment with a high cytokine concentration due to IBMIR in the liver [27]. Currently, an islet volume of 5000 IEQ/kg is commonly required for successful islet transplantation; however, the functional injury of islets can be reduced by regulating the immune response that occurs after transplantation, which is expected to improve transplant outcomes with a smaller amount of islet grafts. In other words, it is not clear to what extent the islet volume is required. In the current study, we demonstrated that BM-MSC and SHED had an immunosuppressive effect on the activity of PBMC via both the expression of PD-L1 and the production of humoral factors and that the effect of SHED was overwhelmingly stronger than that of BM-MSC. Interestingly, BM-MSC and SHED were found to enhance the secretion of immunosuppressive factors in the presence of the combination of IFN-γ, TNF-α, and IL-1β, which mimic an IBMIR environment. BM-MSC and SHED suppress the cytotoxicity of human PBMC toward human insulin-producing cells in a cytokine concentration-dependent manner.

BM-MSC have been shown in several experimental models of islet transplantation to elicit angiogenesis, prevent apoptosis, and suppress allograft rejection [8,13,18,28,29]. Nonetheless, there are few reports, especially in humans, on the effect of SHED on islet transplantation and immunomodulation. We reported that the effects of humoral factors secreted from SHED on the proliferation of pancreatic beta cells [15], angiogenesis [16], and neurite outgrowth [17] were stronger than those secreted from BM-MSC; thus, SHED was expected to be more suitable for islet transplantation. BM-MSC and SHED are MSC with different collection sites and have been reported to have many similarities, including fibroblast-like cell morphology, expression of cell surface proteins, and multipotency of adipogenic, osteogenic, and chondrogenic differentiation [30]. In the present study, differences were observed in the degree of enhanced secretion of inhibitory factors after cytokine stimulation. Additionally, we previously reported differences in the types of factors secreted by BM-MSC and SHED [15,17]; thus, the difference might have influenced the stronger immunosuppressive effect of SHED compared to that of BM-MSC in this study. As the expression of cell surface molecules of SHED did not change in response to cytokine stimulation, SHED should be more stable than BM-MSC, which are known to be very sensitive to culture conditions and prone to changes in cell properties and function [31]. Furthermore, SHED are easy to collect and can be safely used in allogeneic transplantation; thus, taken together, SHED could be a more promising candidate for the simultaneous transplantation of tissue stem cells with pancreatic islets than BM-MSC, which have been widely studied and reported. In a recent report, co-culture of SHED and PBMC demonstrated immunosuppressive effects through increased Tregs and reduced TNF-α secretion, suggesting that SHED may exhibit stronger immunomodulatory capacity than BM-MSC, consistent with our study [32].

This study demonstrated that BM-MSC and SHED exhibited PD-L1-mediated cellular immunosuppression, which was significantly enhanced by cytokine stimulation. PD-L1 expression is induced by various inflammatory stimuli, such as IFNs, IL-1β, IL-6, IL-10, IL-12, IL-17, TGF-β, and TNF-α, in several cell types, including multiple cancers and pancreatic islet [33]. The JAK/STAT-IRF1 pathway is a key regulator of IFN-mediated PD-L1 expression in melanoma cells [34], while NF-κB activation plays a crucial role in lipopolysaccharide-induced PD-L1 expression in macrophages [35]. Recent studies have also reported that IFN-γ is a critical activator of PD-L1 expression and upregulates secreted factors from MSC and enhances MSC-mediated immunosuppression [24,36]. Activated PBMC induced by CD3/CD28 have been also reported to enhance PD-L1 and Fas Ligand expression in dental pulp stem cells through both cell-contact dependent and humoral factors such as IFN-γ, TNF-α, IL-2 and IL-6, ultimately triggering a feedback-like mechanism that switches the activated PBMC themselves into an immunosuppressive state [37]. On the other hand, our study demonstrated that direct activation of SHED and MSCs with inflammatory cytokines enhanced PD-L1 expression and production of humoral factors. This finding indicates that prior activation can efficiently induce immunosuppressive effects and may contribute to achieving stable therapeutic outcomes in clinical applications. Furthermore, MSC secrete soluble PD-L1 ligands, including PD-L2, which are essential for directly modulating the immunosuppressive effects of MSC on T cell behavior [24]. SHED was also reported to ameliorate Sjögren's syndrome by secretion of soluble PD-L1 [38]. It was reported that PD-L1 may not be essential for the immunomodulatory effects of MSC, and the direct role of PD-L1 in MSC-mediated immunosuppression remains controversial [39]. The current study demonstrated the cellular immunosuppressive effects of BM-MSC and SHED through the experiment of inhibiting cell contact. Additionally, PD-L1 expression in BM-MSC and SHED was significantly upregulated several hundred-fold by inflammatory stimuli. The immunosuppressive effect was attenuated in a concentration-dependent manner by PD-L1 antibodies, indicating that PD-L1 may be involved in the cellular immunosuppressive mechanisms of BM-MSC and SHED. Moreover, it was also reported that human islet-like organoids generated from iPS cells could survive and maintain their function for a long time after transplantation into diabetic mice by expressing PD-L1 through IFN-γ stimulation [40]. Based on these results, it is expected that long-term engraftment and functional maintenance of islet-like organoids can be expected in future islet transplantation by stimulating both SHED cells and islets to express PD-L1 through cytokine stimulation. However, PD-L1 blockade experiments showed that PD-L1-mediated immunosuppression was only partial, thus the mechanisms of other cell-to-cell immunosuppression, including HLA-G [41] and Galectin-9/Tim-3 pathways [42], need to be investigated in the future.

To improve outcomes in islet transplantation, the simultaneous transplantation of mesenchymal stem cells and islets has gotten attention at the research level for its various effects, including immunosuppression, functional protection of transplanted islets, and angiogenesis [43,44]. In future clinical applications, with regard to the timing of transplantation of SHED and pancreatic islets, pre-transplantation of SHED into the liver prior to islet transplantation is unlikely to be suitable because transplantation of any type of cell into the liver can trigger liver-specific IBMIR. Thus, SHED and islets should be transplanted simultaneously for clinical applications. Furthermore, to immediately suppress the immune response by secreting factors and cell contact through the PD-1/PD-L1 pathway, it would be better to administer SHED in a pre-activated state. In this study, SHED proved to have sufficient immunosuppressive effects through humoral factors and cell-to-cell attachment with cytokine pre-stimulation; thus, simultaneous transplantation of pre-stimulated SHED and pancreatic islets should be more beneficial in clinical applications. Moreover, to maximize the immunosuppressive effect of simultaneous transplantation of SHED and pancreatic islets, we are trying to create organoids by reaggregation of dispersed human pancreatic islet cells and SHED, even though it is well known that dispersion of pancreatic islets results in impairment of insulin secretory capacity. The reaggregated organoids of pancreatic islet cells and SHED improved the impaired insulin secretory capacity in the present study, and the protective effect was in agreement with that previously reported [25]. Furthermore, it might be easier to efficiently transfer beneficial genes for pancreatic beta cells, such as Mycl that we previously reported that induction of Mycl reprogrammed and expanded functional pancreatic beta cells [45], compared to direct transfection to pancreatic islets without dispersion. These data suggest that organoids containing SHED and dispersed islet cells may be useful for future islet transplantation.

A limitation of the present study is that the immunosuppressive effects of SHED were not investigated *in vivo*. Additionally, the cytotoxicity of PBMC was examined only in human iPS cell-derived pancreatic beta-like cells and not in human pancreatic islets. Although MSC have been reported the cytoprotective effects by inducing pancreatic beta cell maturation [46,47] and reducing endoplasmic reticulum stress [48], the molecular mechanisms of enhanced insulin secretion in islet-like organoids reaggregated from human islet cells and SHED were not evaluated in this study. Further studies are needed to clarify the immunosuppressive and cytoprotective effect of SHED on islet transplantation through cytotoxicity experiments using human islets and *in vivo* using large animals, such as porcine.

The present study showed that SHED had immunosuppressive effects on human PBMC through humoral and cell-contact factors, which were enhanced under cytokine stimulation and were more potent than BM-MSC. Furthermore, SHED improved insulin secretion capacity in reaggregated human pancreatic islets, suggesting that it could be a promising adjunctive therapy for islet transplantation in the future.

## Resource availability

Data are available from the corresponding author upon reasonable request.

## Author contributions

S.T. designed study, researched data and wrote the manuscript. M.K. researched and visualized data and contributed to discussion. K.I. researched and visualized data and contributed to methodology. Y.M. researched data and contributed to methodology. Y.H. researched data. T.I. provided SHED and contributed to methodology. A.Y. provided SHED and contributed to methodology. T.H. contributed to methodology. M.K. contributed to methodology. J.N. reviewed/edited manuscript. T.K. reviewed/edited manuscript. H.K. reviewed/edited manuscript. K.I. designed study, researched and visualized data, contributed to discussion and reviewed/edited manuscript.

## Declaration of competing interest

H.K. received clinically commissioned/joint research grants from EA Pharma Co., Ltd., Otsuka Pharmaceutical Co., Ltd., Kyowa Kirin Co., Ltd., Kowa Company, Ltd., Daiichi Sankyo Co., Ltd., Sumitomo Pharma Co., Ltd. Takeda Pharmaceutical Co., Ltd., Teijin Pharma Ltd., Eli Lilly Japan K.K., Nippon Boehringer Ingelheim Co., Ltd.

S.T., M.K., K.I., Y.M., Y.H., T.I., A.Y., T.H., M.K., T.K. and K.I declare no conflict of interest.

## Data Availability

Data will be made available on request.
